# A Multicenter Propensity Score Matching Study of Homologous Convective Volume (HCV): A New Comprehensive Parameter for Dialysis Dose That Improves Survival

**DOI:** 10.7759/cureus.96157

**Published:** 2025-11-05

**Authors:** Jorge Quinchuela, Gabriela Tamayo, Natalia Benavides, Franklin Mora-Bravo

**Affiliations:** 1 Department of Nephrology, DaVita Ecuador, Unidad Dialcentro, Quito, ECU; 2 Department of Nephrology, DaVita Ecuador, Unidad Sermens, Quito, ECU; 3 Department of Nephrology, DaVita Ecuador, Unidad Dialibarra, Ibarra, ECU; 4 Hemodiafiltration Unit, Pafram, Sucúa, ECU

**Keywords:** hemodiafiltration, hemodialysis, homologous convective volume, mortality, propensity score matching (psm)

## Abstract

Introduction: The evaluation of hemodiafiltration (HDF) effectiveness concerning patient survival has primarily centered on convection volume. A limitation of this approach is that it overlooks two parameters: Kt/V and extracorporeal blood flow (Qb). This study aims to use the homologous convective volume (HCV) to compare mortality rates between patients undergoing hemodialysis and hemodiafiltration, with the hypothesis that higher HCV leads to lower mortality.

Methods: This observational study was conducted across 14 hemodialysis clinics within the Davita-Ecuador group. Patients who had undergone conventional hemodialysis or HDF were included in the study. HCV was calculated as: convective volume * Kt/V * Qb, expressed in liters per minute. Group A included patients with 0 to 9.9 liters of HCV, Group B included patients with 10 to 17.9 liters of HCV, and Group C included patients with approximately 18 or more liters of HCV. The variables analyzed included epidemiological, clinical, laboratory, impedance, medication use, and mortality. To adjust for potential confounders, propensity score matching (PSM) was performed. For group comparisons, ANOVA and Kaplan-Meier survival analysis were used.

Results: Using propensity score matching, 2537 patients were assigned to Group A, 213 to Group B, and 476 to Group C. The three groups were matched based on age, Charlson comorbidity index, sex, and presence of type 2 diabetes mellitus, ensuring equal distribution across the groups. There were no differences in weight distribution, body mass index, or comorbidities among the groups. Survival was 46.9 ± 0.7 months in Group A, 57.8 ± 2.0 months in Group B, and 61.9 ± 1.3 months in Group C (Log Rank (Mantel-Cox) Chi Square 67.65, P<0.001).

Conclusion: HCV is a new and comprehensive parameter for measuring total dialysis dose because it combines the removal of small solutes (Kt/V), the removal of medium-sized solutes (convective volume), and Qb. A higher dialysis dose, as indicated by HCV, is directly linked to increased patient survival. The group receiving the highest dose (HCV of 18 liters or more) showed the greatest longevity (61.9 months) compared to groups with lower doses.

## Introduction

Several studies have indicated that online hemodiafiltration (OL-HDF) may improve survival compared to high-flux hemodialysis (HD) [1-5]. Detailed analysis shows that patients undergoing OL-HDF experience lower all-cause mortality, fewer cardiovascular deaths, and better anemia management. Additionally, studies report decreased use of erythropoiesis-stimulating agents (ESAs), improved phosphorus control, and a lower incidence of beta-2 microglobulin-related amyloidosis [5-7]. OL-HDF has also been linked to better nutritional status, fewer complications, and fewer hospitalizations, all of which contribute to an improved quality of life for dialysis patients. Given that mortality rates in dialysis patients remain high, ranging from 15% to 20% annually, and that increased urea removal has not shown significant direct effects on survival, the focus has shifted toward convective therapies like OL-HDF. Recent randomized controlled trials comparing conventional hemodialysis with online postdilution hemodiafiltration have produced mixed, though mostly positive, results regarding OL-HDF's benefits on mortality and morbidity [8].

Evaluation of the effectiveness of OL-HDF on patient survival has mainly focused on convection volume (Vc), defined as the sum of ultrafiltration volume (Vuf) and infusion volume (Vinf). Previous research has shown that convection volumes greater than 23 liters are associated with better survival rates than volumes less than 23 liters [9]. A limitation of this approach is that it does not include two essential parameters: Kt/V and extracorporeal blood flow (Qb). Kt/V directly relates to the removal of low-molecular-weight solutes, whereas extracorporeal blood flow influences the clearance efficiency of all uremic toxins, regardless of molecular weight.

The omission of these factors in most comparative studies of hemodialysis and hemodiafiltration creates an imbalance in the evaluation of dialysis dose. Relying solely on convection volume may underestimate the overall impact of the total dialysis dose on conventional hemodialysis and, at the same time, overestimate the role of convection clearance in hemodiafiltration without considering the effects of Kt/V and blood flow.

We propose introducing a new unified parameter, the homologous convective volume (HCV), defined as:

HCV=Vc x VKt​ x Qb​

This index combines Kt/V solute dialysis dose, Qb as a measure of total transport flux, and Vc as an indicator of the effectiveness of medium molecular weight solute clearance. By integrating these three parameters, the new index allows for a more precise and fair comparison between conventional hemodialysis and hemodiafiltration, providing a comprehensive metric that reflects the total dialysis dose delivered to the patient. This study aims to use the HCV to compare the mortality rates of a group of patients on hemodialysis and hemodiafiltration, with the hypothesis that higher HCV results in lower mortality.

## Materials and methods

Study design

This study is observational, and its source is prospective.

Scenery

The study took place at 14 hemodialysis clinics in Ecuador that are part of the DaVita Ecuador group. The study period lasted from September 3, 2018, to March 30, 2022.

Participants

Patients who had undergone traditional hemodialysis for more than three months and attended three weekly four-hour sessions were included in the study. The exclusion criteria were patients with COVID, those who did not follow the established treatment schedule (four hours per session, three times a week), and patients whose causes of death were unrelated to chronic kidney disease (CKD) (such as treatment abandonment or referral to kidney transplantation).

Homologous convective volume groups

The protocol for measuring convective volume in both hemodialysis and hemodiafiltration was to sum the infusion and ultrafiltration volumes in liters. In hemodialysis, the infusion volume is zero, so the convective volume equals the ultrafiltration volume. The convective volume was standardized based on the KT/V received and the extracorporeal flow administered during each treatment, allowing comparison across treatments with different convective volumes and Kt/V and Qb prescriptions. It is calculated by multiplying the convective volume, Kt/V, and the extracorporeal flow expressed in liters per minute. The result is given in homologous convective liters (liters*KT/V*liters per minute of Qb). The sample was divided into three groups based on tertiles of HCV: Group A from 0 to 9.9 liters HCV, Group B from 10 to 17.9 liters HCV, and Group C with 18 liters HCV or more. For example, for a case with 24 liters of convective volume, a Kt/V of 1.80, and an average Qb of 0.38 liters per minute, the HCV is calculated as HCV = 24 * 1.8 * 0.38 = 16.416 liters.

Variables

The independent variables included age, sex, dry weight, body mass index, Charlson comorbidity index (CCI), and probability of survival (age-adjusted CCI (ACCI) + albumin). The presence of comorbidities encompassed coronary artery disease, heart failure, peripheral vascular disease, cerebrovascular disease, dementia, chronic lung disease, connective tissue disorders, gastrointestinal bleeding, and liver disease. The etiology of chronic kidney disease featured glomerulonephritis, diabetic nephropathy, polycystic kidney disease, interstitial nephritis, and vascular disease with arterial hypertension. Hemodialysis modalities included OL-HDF and HD, along with Qb, dialysate flow (Qd), Kt/V, convective volume, and ultrafiltration. Pre- and postdialysis blood pressure were measured. Laboratory parameters included potassium, hemoglobin, ferritin, C-reactive protein, albumin, normalized protein catabolism rate (nPCR), phosphorus, calcium, and parathyroid hormone (PTH). Medications administered included erythropoietin and calcitriol. Predialysis relative overhydration was assessed via bioimpedance, as were predialysis lean tissue and the fat tissue index. The dependent variable was mortality.

Data sources/measurements

The source was direct. Data were collected via the EuCliD computer system following patient privacy and consent protocols. The collected data are presented as individual averages. Treatments were performed via Fresenius Medical Care (Bad Homburg, Germany) supplies; the hemodiafiltration machines included 83 Fresenius Medical Care 5008/S volumetric units and 528 Fresenius Medical Care 4008/S hemodialysis machines. FX 60, 80, and 100 Classix dialyzer filters were used for hemodialysis, and CorDiax along with CorAL 600, 800, and 1000 filters were employed for HDF. Bioimpedance readings were standardized at all sites with Body Composition Monitor (BCM) measurements from Fresenius Medical Care. Because parameters such as hemoglobin, potassium, protein C, and PTH can fluctuate monthly, the average of the last quarter was taken at the close of the study.

Propensity score matching

To adjust for potential confounders, propensity score matching (PSM) was performed among the three groups. For PSM, four baseline variables were chosen. The risk of death is strongly linked to age, gender, and diabetes, so these factors were included in the PSM. Additionally, we matched for CCI, albumin, PCR, and lean tissue index, since comorbid conditions can significantly impact mortality. The 1:2:5 PS matched cohorts (low, medium, and high HCV) were created using the nearest-neighbor method with a calliper of 0.01. The balance of baseline variables was evaluated based on standardized mean differences (SMD) before and after PSM. Variables with SMDs below 0.10 following matching were deemed well-balanced. Baseline differences for the covariates included in the propensity score model were also assessed.

Assignment to hemodiafiltration

Hemodiafiltration treatment was assigned to patients based on one of the following criteria: congestive heart failure, recurrent intradialytic hypotension, difficult-to-control hyperphosphatemia, challenging arterial hypertension, and borderline low-flow access. All patients remained on the treatment modality for the entire duration of the study.

Biases

Observation and selection bias were avoided by applying the participant selection criteria. A medical representative from each coordinating center was assigned to compile the data, which was completed using a single online form. The principal investigator consistently maintained the data according to a guide and records approved in the research protocol to prevent potential interviewer, information, and recall bias. When there was any uncertainty about the standard deviation of the data, reviews were conducted through onsite examinations of abnormal data. Two researchers independently analyzed each record in duplicate, and the variables were entered into the database after confirming their consistency.

Study size

The sample was probabilistic. Ecuador has a population of 17,980,083 (2023), with a CKD prevalence of 21,394 cases by 2022. Using EPI Info TM (CDC, Atlanta, GA, USA; Version 7.2.6, October 2023) with an expected mortality rate of 15.7%, a margin of error of 5%, and a 95% confidence level, the minimum sample size was 201 cases in each group.

Quantitative variables and missing data

The results are presented as frequencies and percentages. A scale variable, HCV, was converted into a categorical variable. Missing data was handled by removing 14.13% of the data after propensity score matching. The removed data included 531 cases with disparities in the propensity variables: weight, age, and presence of diabetes.

Statistical analysis

Qualitative variables were analyzed using frequencies and percentages. Proportions were compared with the chi-square test, and means were compared with Student’s t-test. For scaled values, the means of each group were compared using ANOVA. Since some values have statistically significant differences but no clinically relevant differences, the values are differentiated with Cohen's d. Values less than 0.8 are considered to indicate similar distributions with no statistical difference; values greater than 0.8 indicate statistically different distributions. For categorical variables, percentages were compared using the Chi-square test. A survival analysis was conducted with Kaplan-Meier. The statistical package used was SPSS Statistics for Windows version 31.0 (IBM Corp., Armonk, NY, USA).

## Results

**Figure 1:**
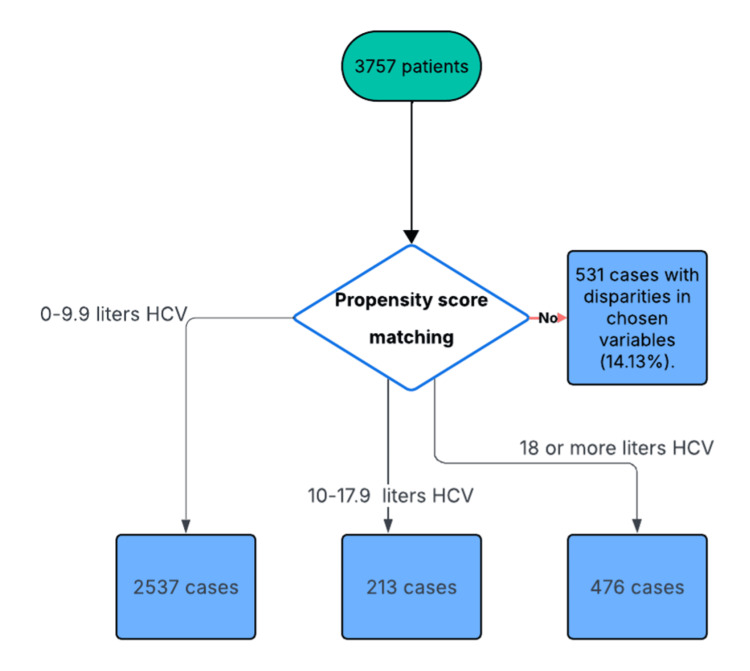
Flow chart of the participants. HCV: homologous convective volume.

A total of 2537 patients in Group A, 213 patients in Group B, and 476 patients in Group C were analyzed (Figure 1).

Main characteristics of the study groups

The three groups were matched by age, CCI, sex, and the presence of type 2 diabetes mellitus through propensity score matching, ensuring the distribution is equal across the groups. There were no differences in weight distribution, body mass index, or the probability of survival at one year among the groups (Table 1). Additionally, there were no differences in the distribution of comorbidities among the three groups (Table 2). Fistulas in Group A were 1565 (62.2%), in Group B 149 (70.3%), and in Group C 426 (89.5%). Prostheses in Group A were 374 (14.9%), in Group B 38 (17.9%) and in Group C 46 (9.7%). Catheters in Group A were 576 (22.9%), in Group B 25 (11.8%) and in Group C were four (0.8%). Vintage in Group A was 17.8 months (SD 21.2), in Group B was 26.9 months (SD 22.1), and in Group C was 29.5 months (SD 21.5).

**Table 1:** General characteristics of the groups, variables in scale. F-value for ANOVAs was used. Cohen's d was used to measure the effect size, quantifying the magnitude of the difference between two means, expressed in units of standard deviation. *d-Value less than 0.80 the groups are the same, greater than 0.80 the groups are different. HCV: homologous convective volume. ACCI Albumin: Index Value Survival Probability Based on Age-Adjusted Charlson Comorbidity Index + Albumin: 1 Year.

	Group A 0-9.9 Liters HCV N=2537	Group B 10-17.9 Liters HCV N=213	Group C 18 or more liters HCV N=476	Cohen’s d A vs B *d-value	Cohen’s d A vs C d-value	Cohen’s d B vs C d-value	F (ANOVA)
Age (Years)	59.3 ± 11.2	59.8 ± 13.8	59.8 ± 12.0	0.040 (0.045)	0.043 (0.045	0 (0)	0.679
Dry Weight (Kg)	63.6 ± 13.8	68.0 ± 14.3	63.5 ± 13.3	0.31 (0.32)	0.007 (0.007)	0.33 (0.315	10.487
Body Mass Index (kg/m^2^)	25.7 ± 4.9	27.0 ± 4.9	25.8 ± 4.8	0.265 (0.265)	0.02 (0.02	0.34	7.215
Charlson Age-Adjusted Index	5.2 ± 1.9	5.2 ± 1.9	5.1 ± 1.9	0.04	0.23	0.20	3.481
ACCI Albumin	75.1 ± 23.0	78.9 ± 20.3	76.9 ± 23.3	0.18	0.19	0.02	3.481
Number of comorbidities	2.0 (1-2)	2 (1-2)	2 (1-2)	0.06	0.05	0.02	0.645

**Table 2:** General characteristics of the groups, nominal and categorical variables. Chi square was used to calculate the p-values. HCV: homologous convective volume.

	Group A 0-9.9 Liters HCV N=2537	Group B 10-17.9 Liters HCV N=213	Group C 18 or more liters HCV N=476	P	Chi-Square
Sex Female	1095 (43.2%)	92 (43.2%)	208 (43.7%)	0.977	0.047
Type 2 diabetes mellitus	1278 (50.4%)	107 (50.2%)	239 (50.2%)	0.997	0.005
Vascular disease / Hypertension	695 (27.4%)	74 (34.7%)	138 (29.0%)	0.072	5.461
Coronary Heart Disease	330 (13.0%)	29 (13.6%)	72 (15.1%)	0.457	1.567
Congestive heart failure	405 (16.0%)	42 (19.7%)	94 (19.7%)	0.063	5.532
Peripheral Vascular Disease	198 (7.8%)	23 (10.8%)	57 (12.0%)	0.006*	10.229
Cerebrovascular disease	198 (7.8%)	16 (7.5%)	38 (8.0%)	0.977	0.046
Chronic lung disease	123 (4.8%)	17 (8.0%)	19 (4.0%)	0.101	5.163
Gastrointestinal bleeding	107 (4.2%)	11 (5.2%)	24 (5.0%)	0.628	0.963
Liver Disease	77 (3.0%)	4 (1.9%)	13 (2.7%)	0.574	0.996
Neoplasia	58 (2.3%)	5 (2.3%)	13 (2.7%)	0.847	0.345
Connective tissue disorder	39 (1.5%)	8 (3.8%)	10 (2.1%)	0.092	5.931
Dementia or other psychiatric illnes	20 (0.8%)	5 (2.3%)	7 (1.5%)	0.068	6.166
Glomerulonephritis	60 (2.4%)	5 (2.3%)	19 (4.0%)	0.107	10.048
Cystic kidney disease	54 (2.1%)	7 (3.3%)	8 (1.7%)	0.434	1.819

Prescription of hemodialysis and hemodiafiltration treatment

There was no difference in the number of dialyses, volume of dialysate fluid flow (Qd), and ultrafiltration received during the month in each group (Table 3). Qb prescription was higher in Group C (Cohen's d >0.8). The convective volume prescription, Sp Kt/V, was progressively higher in each group, which was the goal of comparison in this study (Cohen's d >0.8) (Table 3).

**Table 3:** Descriptions of treatment, laboratories, medications, bioimpedance, and blood pressure in the groups. F-value for ANOVAs was used. Cohen's d was used to measure the effect size, quantifying the magnitude of the difference between two means, expressed in units of standard deviation. *d-Value less than 0.80 the groups are the same, greater than 0.80 the groups are different. HCV: homologous convective volume, Qb: extracorporeal blood flow, Qd: dialysate flow, nPCR: normalized protein catabolism rate, iPTH: intact parathyroid hormone

	Group A 0-9.9 Liters HCV N=2537	Group B 10-17.9 Liters HCV N=213	Group C 18 or more liters HCV N=476	Cohen’s d A vs B *d-value	Cohen’s d A vs C d-value	Cohen’s d B vs C d-value	F (ANOVA)
Data related to the treatment							
Hemodialysis during the month	11.2 ± 3.3	12.2 ± 2.0	12.0 ± 2.5	0.37	0.20	0.18	22.047
Qb (ml/min)	356 ± 33	365 ± 25	393 ± 23	0.42	1.39*	1.12*	294.172
Qd (ml/min)	482 ± 47	493 ± 22	496 ± 16	0.32	0.37	0.05	25.85
Homologous convective volume (Kt/v*Convective Volume*Qd)	1.70 ± 1.1	15.1 ± 2.0	23.1 ± 3.8	8.22*	7.38*	2.56*	30736.5
Infusion volume (Liters)	0.6 ± 2.0	20.2 ± 3.4	23.9 ± 2.6	5.38*	7.09*	1.22*	27220.3
Convective volume (Liters)	2.5 ± 1.7	22.5 ± 3.6	26.2 ± 2.6	7.56*	11.02*	1.18*	34227.5
Ultrafiltration (ml)	2274 ± 634	2441 ± 594	2452 ± 625	0.42	0.29	0.13	20.822
Sp KT/V	1.85 ± 0.38	1.88 ± 0.27	2.25 ± 0.34	0.06	1.08*	1.39	240.543
Laboratory tests (average of the last 3 months)							
Hemoglobin (g/dL)	10.8 ± 1.3	11.3 ± 1.4	11.1 ± 1.0	0.51	0.25	0.31	26.132
Predialysis potassium (mEq/L)	5.07 ± 0.59	5.10 ± 0.48	5.03 ± 0.48	0.06	0.07	0.15	1.175
Albumin (g/dl)	3.96 ± 0.45	4.05 ± 0.28	4.07 ± 0.27	0.35	0.35	0	18.334
nPCR (gr/kg/d)	0.98 ± 0.19	1.04 ± 0.14	1.04 ± 0.17	0.23	0.28	0.28	21.964
C-reactive protein (mg/L)	28.7 ± 51.2	15.8 ± 18.7	16.4 ± 21.3	0.51	0.25	0.31	19.692
Phosphorus (mg/dl)	4.3 ± 1.2	4.4 ± 1.0	4.2 ± 1.1	0.09	0.12	0.21	3.708
Calcium (mg/dl)	8.7 ± 0.6	8.7 ± 0.5	8.8 ± 0.5	0.04	0.16	0.13	2.580
iPTH (pg/ml)	308 ± 313	363 ± 307	405 ± 439	0.18	0.25	0.11	10.275
Ferritin (ng/ml)	791 ± 434	798 ± 390	892 ± 420	0.07	0.17	0.25	11.192
Dialysis medications							
Erythropoietin alpha (Unit/Kg/week)	85 ± 47	84 ± 46	90 ± 46	0.14	0.09	0.24	0.785
Iron IV (mg/month)	214 ± 66	226 ± 63	209 ± 50	0.11	0.09	0.22	5.109
Number of blood pressure drugs	2.0 ± 0.8	2.0 ± 0.8	2.0 ± 0.8	0.01	0.16	0.17	5.965
Bioimpedance							
Lean tissue index	11.8 ± 2.8	12.1 ± 3.1	12.0 ± 2.6	0.29	0.18	0.11	2.287
Fat tissue index	13.9 ± 5.9	14.7 ± 5.7	13.5 ± 5.3	0.14	0.07	0.22	4.195
Relative overhydration pre-dialysis (%)	13.4 ± 8.8	11.4 ± 7.0	12.5 ± 7.1	0.24	0.17	0.08	6.742
Pre- and post-dialysis blood pressure							
Pre-dialysis systolic blood pressure (mmHg)	147 ± 15	145 ± 13	149 ± 15	0.03	0.10	0.14	6.745
Pre-dialysis diastolic blood pressure (mmHg)	76 ± 7	74 ± 6	76 ± 8	0.10	0.19	0.10	4.034
Post-dialysis systolic blood pressure (mmHg)	142 ± 14	140 ± 11	145 ± 13	0.15	0.15	0.32	9.976
Post-dialysis diastolic blood pressure (mmHg)	74 ± 6	74 ± 5	74 ± 7	0.04	0.05	0.09	1.187

Study of survival

**Figure 2:**
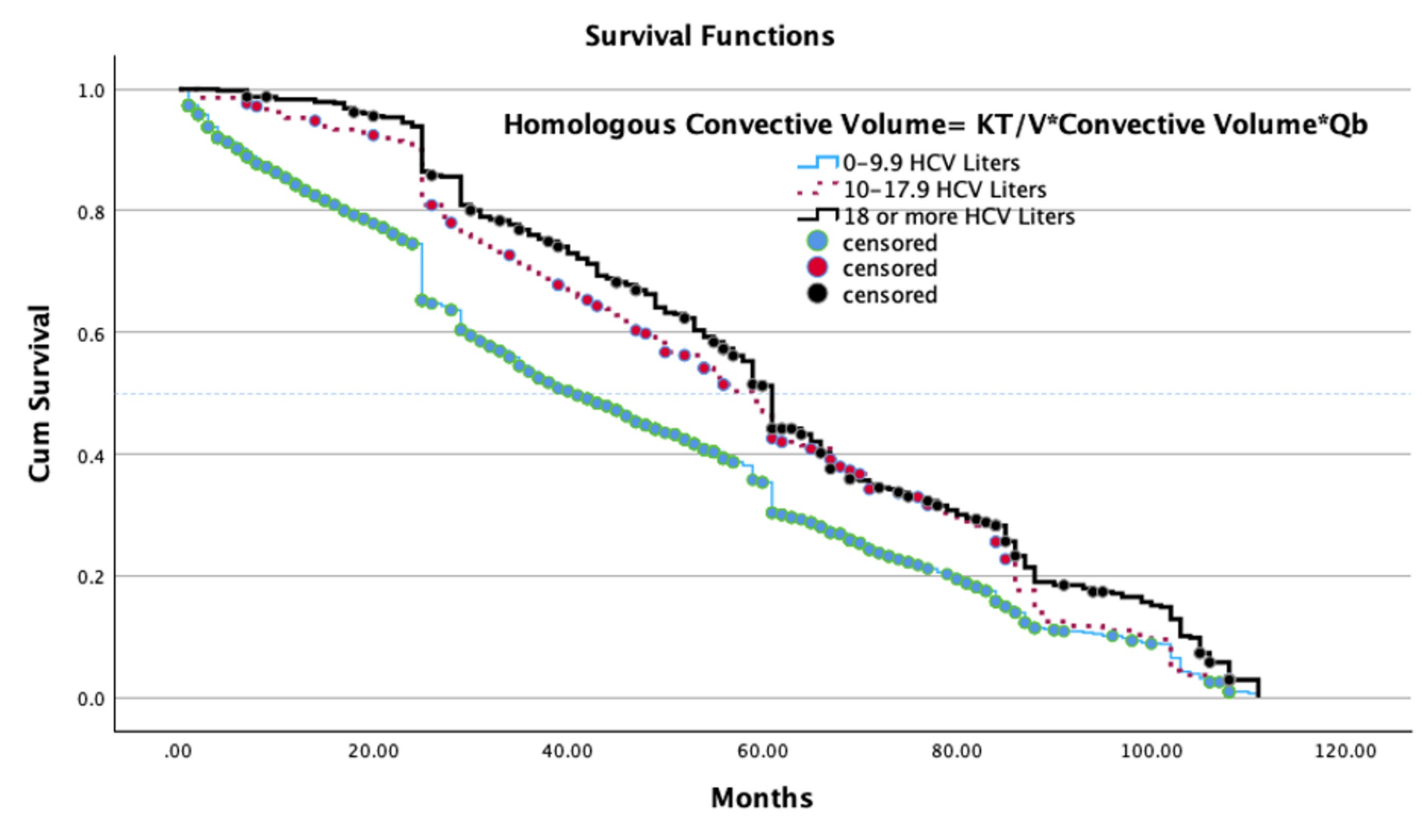
Kaplan-Meier curve HCV: homologous convective liters.

Survival was statistically higher in Group C (61.9 ± 1.3 months), compared to Group B (57.8 ± 2.0 months) and Group A (46.9 ± 0.7 months) (Log Rank Mantel-Cox, Chi Square 67.65, P <0.001) (Figure 2).

The three groups had similar levels of hemoglobin, pre-dialysis potassium, albumin, nPCR, phosphorus, calcium, intact PTH (iPTH), ferritin, and C-reactive protein (Cohen's d <0.8). Dialysis medications, body composition measured by bioimpedance, and blood pressure showed similar statistical distributions across the three groups (Cohen's d <0.8).

## Discussion

Main findings

In the present study, the groups were similar in their basic characteristics, while patient survival was significantly higher in the group that received the highest volume of approved convective therapy (Group C). The three groups (A, B, and C) were comparable in most aspects, such as demographic variables, comorbidities, and laboratory data. The groups also showed similarities in medication, lean and fat tissue composition, and blood pressure values. However, there were notable differences in hemodialysis treatments: convective volume increased progressively in groups B and C compared to Group A, along with significantly higher Qb in Group C, which received the highest number of Kt/V dialysis doses. The most important finding was the impact of treatment on patient survival. Group C, which received the highest approved convective volume (18 or more liters HCV), had the longest survival (61.9 months). Group B (10-17.9 liters HCV) had an intermediate survival (57.8 months), while Group A (0-9.9 liters HCV) had the lowest survival (46.9 months). What is new and novel about this research is that it demonstrates a significant relationship between a new unified parameter - HCV - which considers convection (removal of medium-sized molecules), diffusion (Kt/v: removal of small molecular molecules), extracorporeal flow, and patient survival. This makes the HCV index useful for measuring total dialysis dose.

Interpretations

The rate at which blood (Qb) is processed not only enhances diffusion but also directly provides an adequate convection volume, making it a significant component of the total dialysis dose. The study indicates that higher HCV levels correlate with more prolonged survival. Since HCV incorporates Qb as a component, and Group C (which has the highest survival) had a significantly higher Qb, this suggests that increased blood flow enables a higher dialysis dose, more convection, and thus better clinical outcomes. Traditionally, optimizing dialysis dose has focused mainly on session duration and dialyzer efficiency (via Kt/V). Qb is a clinical factor that may not have received the recognition it deserves in routine practice. The convective volume influences the clearance of medium-sized toxins like B2 microglobulin, alongside Kt/V, which has a small-sized diffusive-toxin clearance, and Qb, which increases both convective and diffusive doses. All three parameters are equally important.

Practical application

Applications in clinical practice include optimizing treatment not only based on Kt/V but also HCV as a more comprehensive target. A higher HCV can be achieved by adjusting Qb and Vc. In practice, blood flow should be viewed as an active variable rather than just a static technical setting. Efforts should focus on ensuring vascular access that supports high and sustained blood flows, such as creating well-caliber arteriovenous fistulas and implementing fistula exercise programs to promote their development and enable high extracorporeal flows of 480 to 500 ml/min.

Related studies

The optimal accepted convective volume is 23 liters, and hemodiafiltration with this volume is known to reduce mortality compared to high-flow hemodialysis [8-10]. However, to date, this is the first study to consider the larger convective volume associated with the higher Kt/V, and the increased extracorporeal flow may improve patient survival. Meanwhile, our study group has been focusing on safe increases in extracorporeal flow based on the dynamic pressure of the arterial line [11]. Each of these factors, when considered in isolation, has been shown to increase survival [12]; together, in this study, they have been demonstrated to have an additive effect on improving survival.

Limitations

Although propensity score matching was used to match the groups by age, sex, and comorbidities, completely eliminating selection bias in such a study remains difficult. Patients in Group C, who received more intensive therapy, might have been chosen for that treatment because they had better vascular access, making them intrinsically more likely to survive. The study cannot account for these factors that could influence the outcomes. There are no data on vascular access flow measurements.

Lines of research

Future research should examine the relationships between the HCV index through randomized studies comparing different extracorporeal high-flow hemodiafiltration doses. It is important to develop programs that gradually increase extracorporeal flow within high therapeutic ranges of 480 to 500 ml/min, and even higher in specific cases.

Generalisability

The study involves a diverse group of Ecuadorian adult patients: 60% mestizos and 40% indigenous people from the Ecuadorian highlands, Afro-Ecuadorians, and Montubios. It includes patients with diabetes mellitus and hypertension, which are common causes of kidney failure worldwide. Patients with disabilities and lower extremity amputations are also part of the study.

## Conclusions

In the present observational study, Group C, with high convective volume more than 17.9 liters HCV adjusted for Kt/V and Qb, had a significantly higher survival of 61.9 months compared to Group B (57.8 months) and Group A (46.9 months), although the three groups were well balanced in terms of baseline characteristics such as age, comorbidities and type 2 diabetes due to propensity score matching. HCV is a new, effective, and comprehensive parameter for measuring the total dialysis dose because it combines Kt/V, the removal of medium-sized solutes (convective volume), and Qb. Additionally, these findings need validation through time-dependent randomized or quasi-experimental studies.
